# Magnetotelluric Signal-Noise Identification and Separation Based on ApEn-MSE and StOMP

**DOI:** 10.3390/e21020197

**Published:** 2019-02-19

**Authors:** Jin Li, Jin Cai, Yiqun Peng, Xian Zhang, Cong Zhou, Guang Li, Jingtian Tang

**Affiliations:** 1 Hunan Provincial Key Laboratory of Intelligent Computing and Language Information Processing, College of Information Science and Engineering, Hunan Normal University, Changsha 410081, China; 2 State Key Laboratory of Nuclear Resources and Environment, East China University of Technology, Nanchang 330013, China; 3 School of Geosciences and Info-Physics, Central South University, Changsha 410083, China

**Keywords:** magnetotelluric, signal-noise identification and separation, approximate entropy (ApEn), multiscale entropy (MSE), stagewise orthogonal matching pursuit (StOMP)

## Abstract

Natural magnetotelluric signals are extremely weak and susceptible to various types of noise pollution. To obtain more useful magnetotelluric data for further analysis and research, effective signal-noise identification and separation is critical. To this end, we propose a novel method of magnetotelluric signal-noise identification and separation based on ApEn-MSE and Stagewise orthogonal matching pursuit (StOMP). Parameters with good irregularity metrics are introduced: Approximate entropy (ApEn) and multiscale entropy (MSE), in combination with k-means clustering, can be used to accurately identify the data segments that are disturbed by noise. Stagewise orthogonal matching pursuit (StOMP) is used for noise suppression only in data segments identified as containing strong interference. Finally, we reconstructed the signal. The results show that the proposed method can better preserve the low-frequency slow-change information of the magnetotelluric signal compared with just using StOMP, thus avoiding the loss of useful information due to over-processing, while producing a smoother and more continuous apparent resistivity curve. Moreover, the results more accurately reflect the inherent electrical structure information of the measured site itself.

## 1. Introduction

Since the Soviet scholar, Tikhonov, and the French scholar, Cagniard, proposed the magnetotelluric (MT) method in the early 1950s [1,2], with its unique advantages, it has gradually been regarded by geophysicists as an important and indispensable method for geophysical exploration. As it is a kind of electrical branch method for sounding by changing the frequency of the electromagnetic field, the exploration depth varies with the frequency of the electromagnetic field. The shallowest depths can be tens of meters while the deepest depths can be up to hundreds of kilometers. It has been widely used in many fields, such as the survey and exploration of petroleum and natural gas, the investigation of geothermal fields, and the prediction of natural earthquakes, etc. [3,4,5,6]. In addition, it is used to study the electrical structure of the crust and upper mantle, in which the discovery of the highly conductive layer provides an important basis for tectonic mechanics.

A direct problem faced is that natural magnetotelluric signals are extremely weak and are susceptible to various types of noise pollution. Performing effective signal-noise identification and the separation of magnetotelluric signals [7,8] has always been a challenge. However, the effectiveness of processing directly affects further analysis of magnetotelluric signals. So far, there have been many methods for the processing of magnetotelluric signals. Most of them are overall processing and, as they lack signal-noise identification, they can easily cause the over-processing of signals and a loss of useful information. Taking this into account, this paper introduces the k-means clustering algorithm, approximate entropy (ApEn), and multiscale entropy (MSE) as the signal-noise identification method. 

The k-means clustering algorithm [9,10,11] is a hard clustering algorithm, which is representative of a typical prototype-based clustering method for objective functions. It utilizes the distance from data points to the prototype as an optimized objective function and an adjustment rule of iterative operation is obtained by using the method of function extreme value evaluation [12,13,14,15]. Entropy is a method of measuring and quantitatively describing the randomness or irregularity of a time series or nonlinear signals [16]. Pincus first proposed the ApEn method in 1991 [17], and Richman and Moorman then proposed the sample entropy (SampEn) [18]; both have the advantages of minimal data requirements, strong anti-noise ability, etc. However, SampEn is a measure of the irregularity of a time series on a single scale, whereas Costa et al. developed another method, MSE, based on the sample entropy [19,20]; it is used to measure the irregularity of time series at different scales and greatly enhances the applicability of entropy [21,22,23]. The above values of entropy can be used as indicators and characteristic parameters to characterize the randomness or irregularity of different types of signals [24,25]. This paper will demonstrate how ApEn and MSE, in combination with k-means clustering, can be used to accurately identify weak magnetotelluric signals and noise interference. Based on ensuring the accuracy of signal-noise identification, the loss of useful information is avoided. In recent years, Donoho et al. proposed a new theory of information acquisition known as compressed sensing [26,27]. Compressed sensing theory mainly includes the sparse representation of signals, an observation matrix measurement, and a reconstruction algorithm. Stagewise orthogonal matching pursuit (StOMP) belongs to the class of greedy reconstruction algorithms in compressed sensing theory [28,29]. It is an improved algorithm for matching pursuit (MP) [30,31] as well as orthogonal matching pursuit (OMP) [32,33]. Compared with MP and OMP, it can further improve the computational efficiency, reduce the computational cost, and improve the de-noising performance. Thus, we propose a novel method of magnetotelluric signal-noise identification and separation based on ApEn-MSE and StOMP.

Although ApEn-MSE does not seem to be directly related to StOMP, they are two indispensable components of the magnetotelluric signal-noise identification and separation method. ApEn-MSE is used as part of the magnetotelluric signal-noise identification, and StOMP is used for the magnetotelluric signal-noise separation. Without the previous signal-noise identification, the subsequent signal-noise separation will have no target.

## 2. Methods

### 2.1. Approximate Entropy (ApEn)

ApEn is a measuring method that quantitatively describes the irregularity of nonlinear signals [34,35,36,37]. Taking the time series {u(i)/i=1,2,⋯,N} of length N as an example, the regularity of u(i) can be measured by approximate entropy in a multi-dimensional space. The approximate entropy is calculated as follows:

(1) Reconstruct the m-dimensional vector, R(i), according to the sequence, {u(i)}:

(1) R(i)=(u(i),u(i+1),⋯,u(i+m−1)), i=1,2,⋯,N−m+1

(2) Calculate the distance, d(R(i),R(j)), between the elements of vector R(i) and R(j):

(2) $$d\left(R\left(i\right),R\left(j\right)\right)=\underset{k=0,1,\cdots ,m-1}{\max}\left\{\left|u\left(i+k\right)-u\left(j+k\right)\right|\right\}\;i,j=1,2,\cdots ,N-m+1$$

(3) For each i value and a measure of similarity, λ(λ>0), the number of vectors, R(j), satisfying the condition, d(R(i),R(j))<λ, is calculated. Additionally, the ratio of this number with respect to the total N−m+1 is called $C_{i}^{m}\left(\lambda \right)$:

(3) $$C_{i}^{m}\left(\lambda \right)=\frac{\begin{array}{cc} number & of \end{array}\left\{d\left(R\left(i\right),R\left(j\right)\right)<\lambda \right\}}{N-m+1}$$

(4) The similarity, $C_{i}^{m}\left(\lambda \right)$, in the above formula is taken as a logarithm, and the average value of i is obtained: $\Phi^{m}\left(\lambda \right)=\frac{1}{N-m+1}\sum_{i=1}^{N-m+1}\ln C_{i}^{m}\left(\lambda \right)$; by increasing the dimension to m+1 and repeating the above step (1) through to (3), $\Phi^{m+1}\left(\lambda \right)$ is obtained. The approximate entropy of the time series, {u(i)}, is defined as:

(4) $$ApEn\left(m,\lambda ,N\right)=\Phi^{m}\left(\lambda \right)-\Phi^{m+1}\left(\lambda \right)$$

The larger the approximate entropy, the more random or irregular the signal will be. 

### 2.2. Sample Entropy (SampEn)

SampEn is a time series complexity measure method, which has the advantages of simple calculation and fast speed [38,39,40,41]. The larger the sample entropy, the more random or irregular the sequence and the lower the self-similarity.

The calculation steps of the sample entropy of the time series, {u(i)}, with length N are similar to the approximate entropy in Section 2.1 and are defined as follows:

(5) $$SampEn\left(m,\lambda ,N\right)=-\ln\frac{B^{m+1}\left(\lambda \right)}{B^{m}\left(\lambda \right)}$$

where λ represents a measure of similarity, $B^{m}\left(\lambda \right)$ represents a logarithmic mean of similarity, and $B^{m+1}\left(\lambda \right)$ represents an m+1-dimensional, $B^{m}\left(\lambda \right)$.

Sample entropy can be used to describe nonlinear signals with a high complexity and a large computational requirement [42,43].

In the experiment, the embedding dimension, m, of the approximate entropy and the sample entropy is taken as 2, while the similarity tolerance, λ, of the approximate entropy and sample entropy is 0.25 times the standard deviation.

### 2.3. Multiscale Entropy (MSE)

MSE is used to calculate the sample entropy on multiple scales of the original signal and it is obtained by using a coarse granulation process at different scales [44,45,46,47].

(1) Take the one-dimensional time series, {u(i)/i=1,2,⋯,N}, as an example. A new coarse grain vector, $\left\{y^{\left(\tau \right)}\right\}$, could be obtained as follows:

(6) yj(τ)=1τ∑i=(j−1)τ+1jτui

where 1≤j≤Nτ, and the scaling factor, τ, is an integer in the $\left[1,2,\cdots ,\tau_{\max}\right]$. 

(2) The sample entropy of τ coarse-grained sequences is also obtained. The MSE analysis is calculated by plotting MSE as a function of the scaling factor, τ.

### 2.4. Stagewise Orthogonal Matching Pursuit (StOMP)

Suppose x is a discrete signal of length N, in the sparse base, $\Psi =\left[\psi_{1},\psi_{2},\cdots \psi_{N}\right]$, only K coefficients are not 0 or significantly larger than other coefficients, and K<<N, we consider that the discrete signal, x, is sparse, also known as K-sparse. The discrete signal, x, can be represented by S on the sparse basis, $\Psi =\left[\psi_{1},\psi_{2},\cdots \psi_{N}\right]$, then $x=\sum_{i=1}^{N}S_{i}\psi_{i}=\Psi S$. M(K<<M<<N) linear projections, $y\left(j\right)=\langle x,\phi_{j}^{T}\rangle$, are obtained on the observation matrix, $\Omega =\left(\varphi_{1}^{T},\varphi_{2}^{T},\cdots \varphi_{M}^{T}\right)$, and j∈{1,2,⋯,M} is used to accurately reconstruct the original signal. The form of the matrix can be expressed as y=Ωx=ΩΨS. The zero norm is actually the number of non-zero elements in the original signal, x, which is the sparsity of x. If x is K-sparse, the zero norm is K. Then, the approximation process of the signal can be equivalent to solving the optimal solution problem under the following constraints:

(7) $$\hat{x}=\arg\min{\left\|x\right\|}_{0}\;subject\;to\;\Omega x=y$$

To further improve the calculation efficiency and reduce the computational cost, the StOMP algorithm is introduced to separate a signal and interference [48]. The signal to be processed must be sparsely represented, and the sparse representation needs to construct the sparse basis. According to the common interference types of magnetotelluric signals, the sparse basis in the paper contains the wavelet packet and cosine atoms. The more similar the selected atom is to the interference itself, the better the signal-noise separation effect. At the same time, the support set is updated and the approximate solution is obtained by using least squares to complete the residual update. Therefore, the algorithm uses the updated residuals to track the matching of atoms, reducing the number of iterations and improving the reconstruction efficiency.

The specific steps of the StOMP algorithm are as follows:

y is the M-dimensional observation vector, Ω is the M×N-dimensional observation matrix, and ${\hat{x}}_{s}$ is the reconstruction signal.

(1) Initialize the residual, $r_{s}=y$, where the counter, s=1;

(2) Calculate the inner product, $C_{s}=\Omega^{T}r_{s}=\langle \Omega ,r_{s}\rangle$;

(3) The set, $J_{s}=\left\{j:\left|C_{s}\left(j\right)\right|>\theta \delta_{s}\right\}$, is generated by setting the soft threshold; here, $\delta_{s}$ is a noise level, $\delta_{s}={\left\|r_{s}\right\|}_{2}\sqrt{M}$, and θ is a threshold parameter;

(4) Update the support set, $I_{s}=I_{s-1}\cup J_{s}$, and use the least squares method to calculate the approximation, $x_{s}$, ${\left(x_{s}\right)}_{I_{s}}={\left(\Omega_{I_{s}}^{T}\Omega_{I_{s}}\right)}^{-1}\Omega_{I_{s}}^{T}y$;

(5) The residual is updated, $r_{s}=y-\Omega x_{s}$;

(6) Check the termination condition. If s>10 or ${\left\|r_{s}\right\|}_{2}<O_{PT}\times {\left\|y\right\|}_{2}$ ($O_{PT}=10^{-6}$), the algorithm terminates and $\overset{\wedge }{x_{s}}=x_{s}$ is the final output. Otherwise, set s=s+1 and we proceed to the above step (2) to continue with the algorithm flow.

## 3. Simulation Analysis

### 3.1. Sample Library Signals Classification

**Figure 1:**
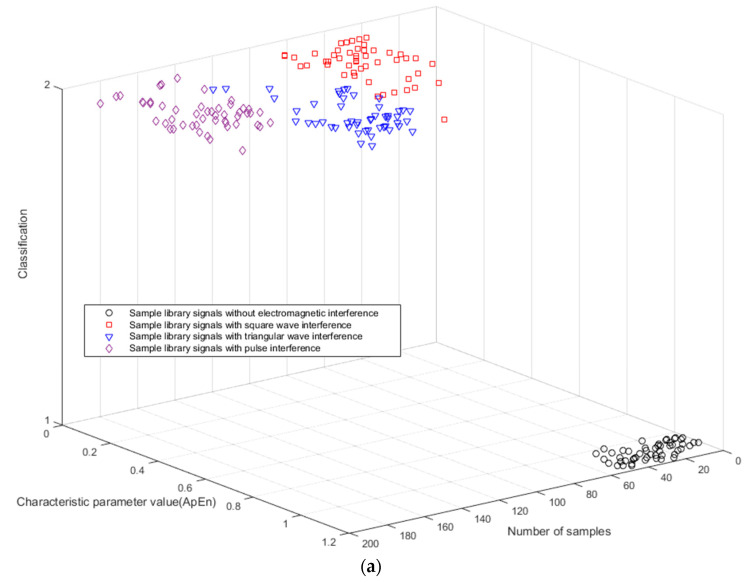

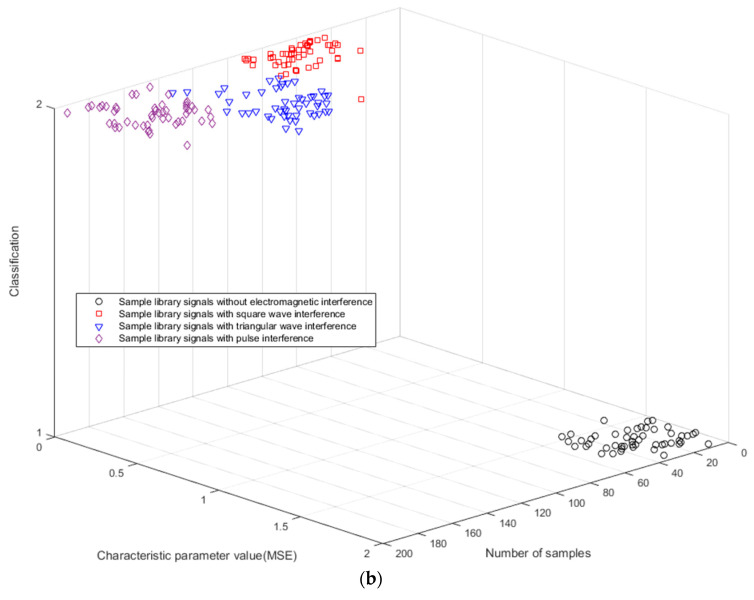
Clustering effect diagrams of sample library signals with (**a**) Approximate entropy (ApEn) and (**b**) Multiscale entropy MSE.

Figure 1 is two 3D clustering effect diagrams of sample library signals [49]. The *x*-axis represents the number of samples. The *y*-axis represents the characteristic parameter values calculated for 200 samples. The *z*-axis indicates that the two different types are formed by extracting the characteristic parameters from the sample library signals as input to the k-means clustering. The first 50 are without electromagnetic interference samples, and the other 150 are samples that are subject to three types of interference (square wave interference, triangular interference, and pulse interference). 

Since the time series of the sample library signals without electromagnetic interference are more random and irregular, as shown in Figure 1, the characteristic parameter value (ApEn or MSE) of MT signals without electromagnetic interference is significantly higher than that of the sample library signals with interference. Moreover, the greater the calculated entropy value, the less interference the signal is subjected to. Therefore, it is feasible to calculate the entropy of the MT signal and input it to the k-means clustering to obtain two different types of signals to realize signal-noise identification.

### 3.2. Add Artificial Interference to the Test Site Signal

**Figure 2:**
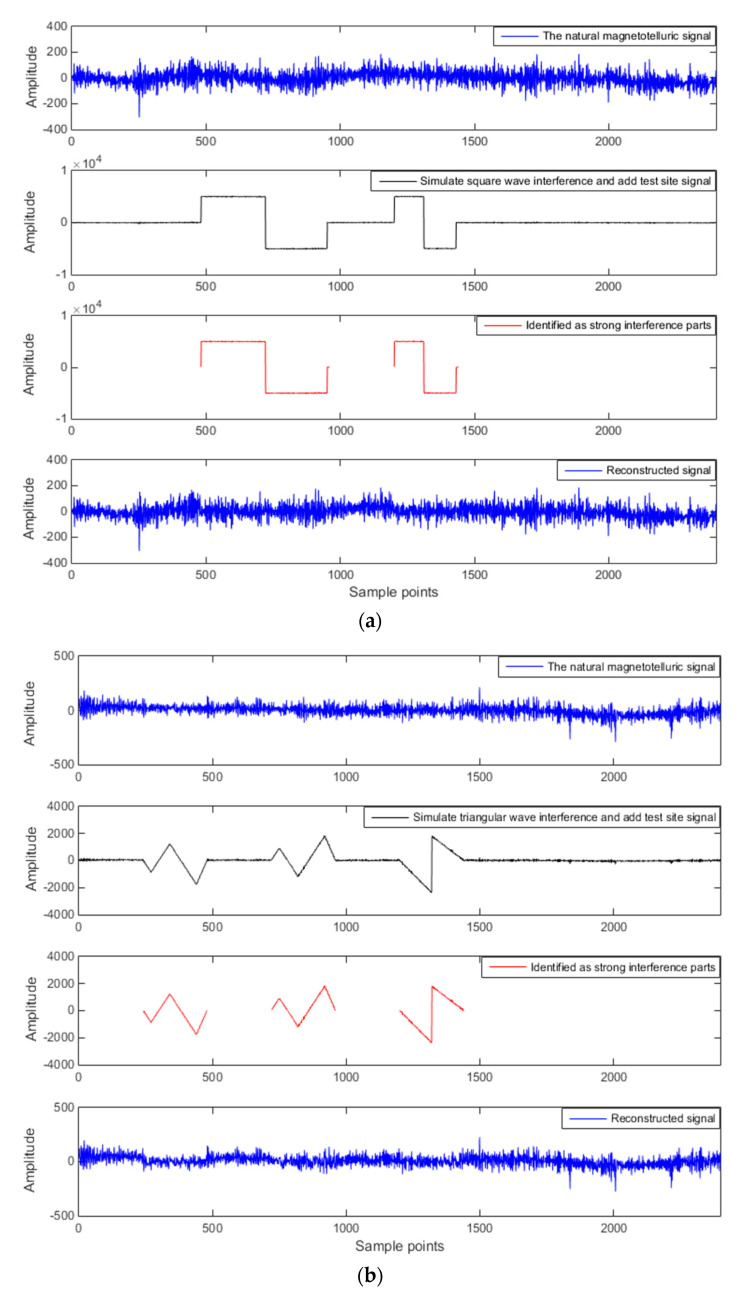

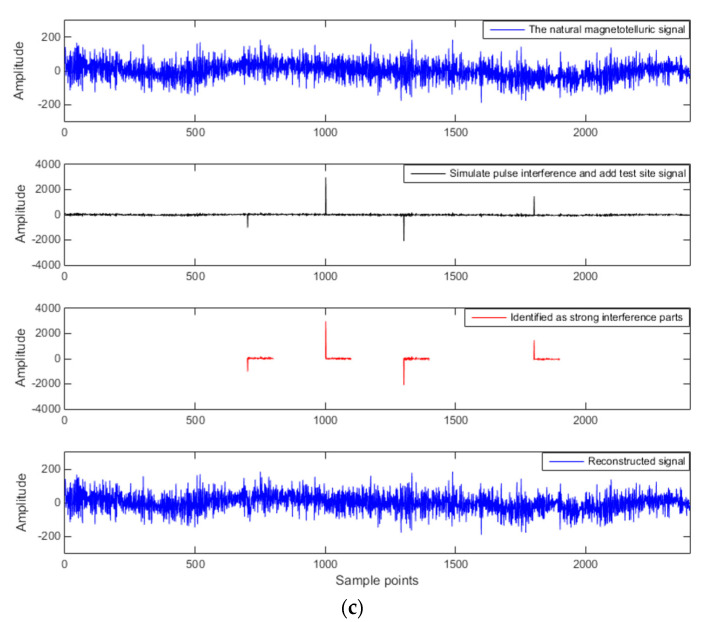
Simulated interference to add to the test site signal with (**a**) square wave interference, (**b**) triangular wave interference, and (**c**) pulse interference.

To verify the effectiveness of the method, simulated large-scale square wave interference, triangular wave interference, and pulse interference are added to the test site signal. The test site is from Qinghai Province, China, located in an area that is far away from any industrial areas, is sparsely populated, and has almost no external noise pollution. The test site signal is the natural magnetotelluric signal, which is what we call a signal that is almost unaffected by noise. The result is shown in Figure 2. In Figure 2, “Sample points” refers to the length of data to be processed. Simulated signals have no temporal resolution and only represent the data length. Figure 2 shows that the approximate entropy and multiscale entropy of the sample library signals, when they are extracted and input to k-means clustering, can be used to accurately identify strong interference and useful signals.

To further illustrate the feasibility of the method proposed herein, normalized cross-correlation (NCC) [50] and signal-noise ratio (SNR) [51] are specifically introduced for the above three types of interference. Table 1 compares the two evaluation indicators of the original test site data and the reconstructed signal.

As can be seen from Table 1, the NCC values between the original test site data and the reconstructed signal are greater than 0.95, and the signal-noise ratios are greater than 10. Therefore, StOMP has a good de-noising effect and can effectively retain the original useful signal.

## 4. Measured Data Analysis

### 4.1. Time Domain Analysis

**Figure 3:**
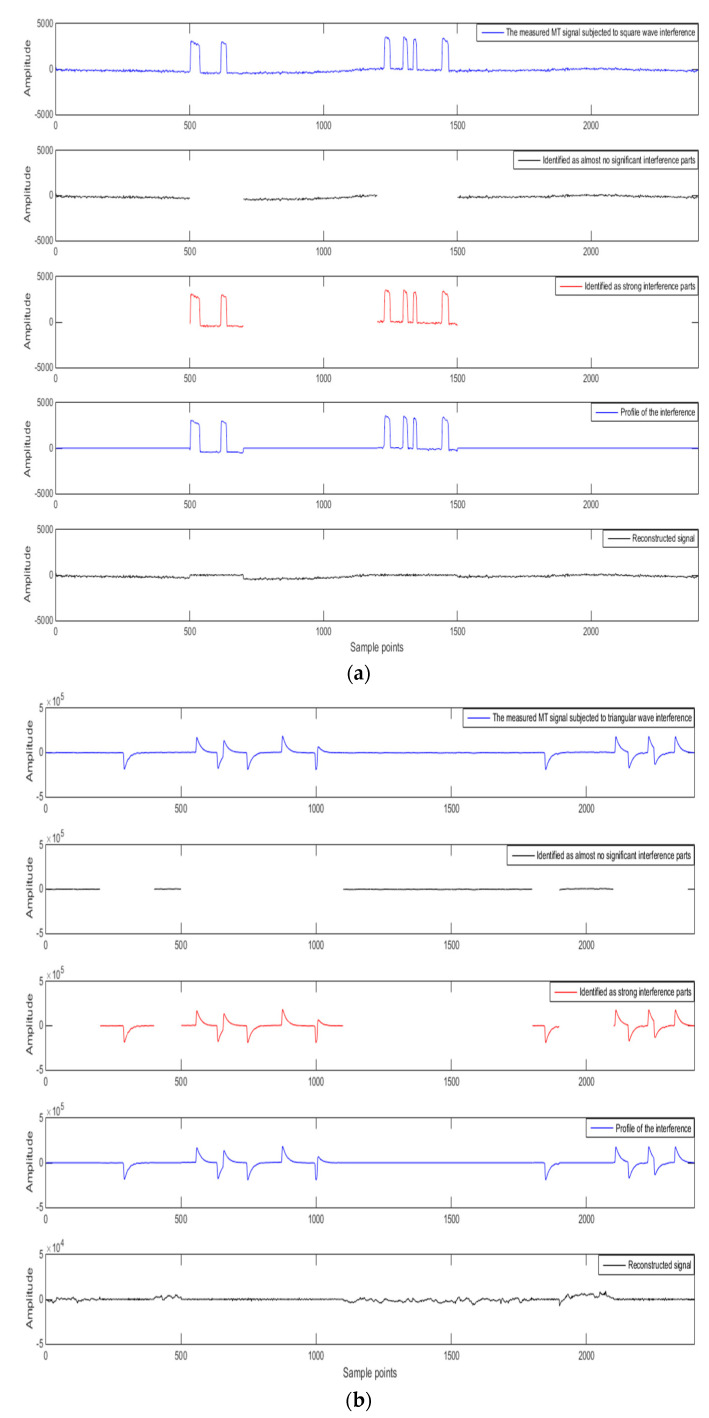

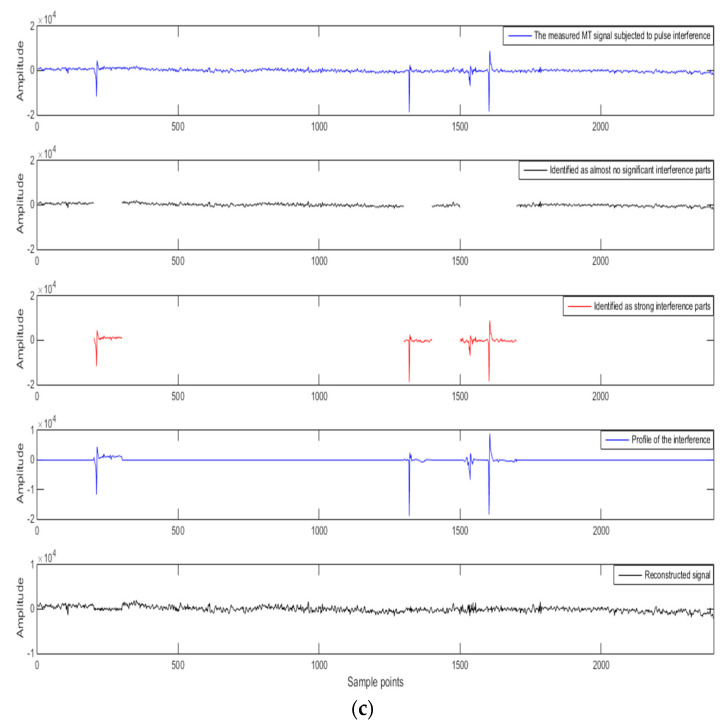
The effect of signal-noise identification and separation for measured magnetotelluric (MT) data with (**a**) square wave interference, (**b**) triangular wave interference, and (**c**) pulse interference.

Figure 3 shows the signal-noise identification and separation effect of measured MT signals subjected to square wave interference, triangular wave interference, and pulse interference. These data are from the Lu-Zong ore-concentration area in Anhui Province, China. For the measured data, the sampling rate is 24 Hz. 2400 sampling points are selected for analysis, that is, data is processed every 100 s. The data are subject to different types and varying degrees of noise interference and they are used to verify the effectiveness of the proposed method. 

Figure 3 shows that the method applied in the measured data processing can still accurately identify the interfered data segment and effectively suppress the interference. This method avoids the loss of useful signals and can better preserve the low-frequency slow-change information. 

### 4.2. Apparent Resistivity-Phase Curve Analysis

As the operating frequency decreases, the depth of exploration will gradually increase. Impedance values at different frequencies measured on the ground can be used to obtain information about the resistivity of the subsurface medium as a function of depth. It can be seen that the apparent resistivity reflects the comprehensive situation of the electrical properties of the rock that can be affected by the influence of the electromagnetic field in a certain frequency range. When the frequency is different, the range of the influence of the electromagnetic field is different, which reflects the resistivity (Ωm) at different depths measuring the signals of different frequencies. Thus, we use the trend of the apparent resistivity-phase curve as an important indicator to evaluate the degree of interference of the measured sites.

**Figure 4:**
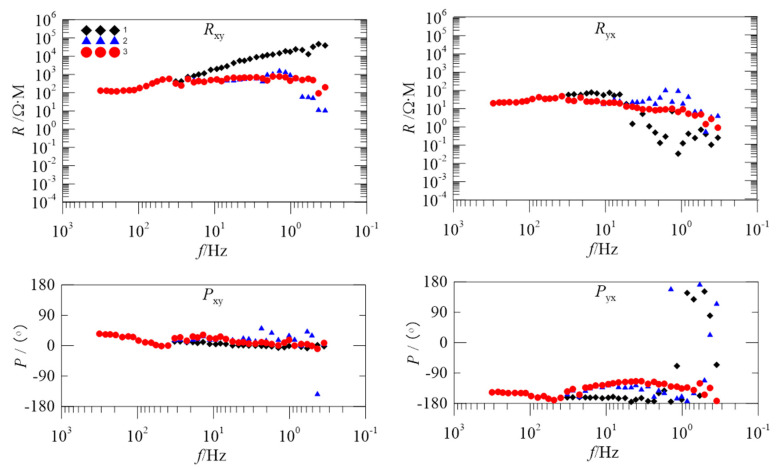
Comparison of the apparent resistivity-phase curves for site 2535BOAC; the black diamonds, blue triangles, and red circles show the apparent resistivity curves obtained from the original data, the overall processing of Stagewise orthogonal matching pursuit (StOMP), and the proposed method, respectively.

**Figure 5:**
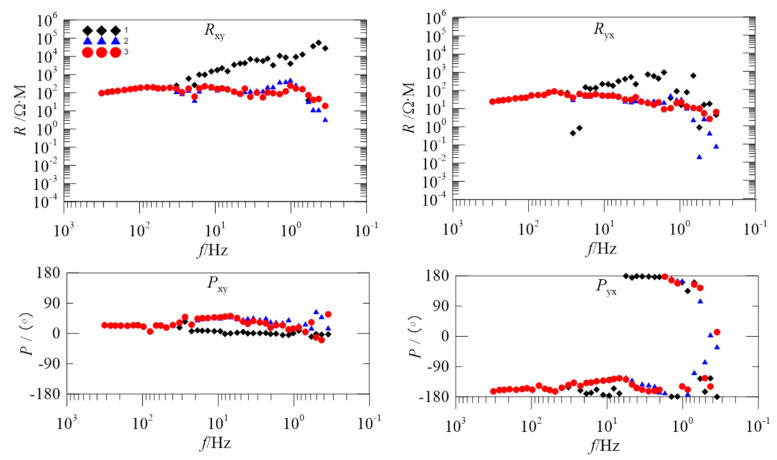
Comparison of the apparent resistivity-phase curves for site 2535BOAF; the black diamonds, blue triangles, and red circles show the apparent resistivity curves obtained from the original data, the overall processing of StOMP, and the proposed method, respectively.

To further evaluate the proposed method, we introduce the data of two measured sites for processing. These two sites are from the Tong-Ling ore-concentration area in Anhui Province, China and are subject to different levels of noise interference. Figure 4 and Figure 5 are the apparent resistivity-phase curves of 2535BOAC and 2535BOAF, respectively.

In Figure 4, the black diamonds are the apparent resistivity-phase curve of the original data. In the 40–0.3 Hz frequency band, the apparent resistivity curve in the $R_{xy}$ direction asymptotically rises to nearly 45°, and the value of the apparent resistivity increases from 100 Ωm to 100,000 Ωm. The above phenomenon is known as a near-source effect. If there is no electromagnetic interference around the measured site, the apparent resistivity and phase of the geological structure reflected in different frequency bands should be relatively stable and should not fluctuate greatly. Due to the presence of various strong electromagnetic interferences, the noise robustness of the low-frequency band data is obviously worse and thus the result cannot truly reflect the information of a deep underground structure. 

The blue triangles are the apparent resistivity-phase curve after being processed just using the StOMP algorithm. Although the trend of asymptotic rising of the apparent resistivity curve in the $R_{xy}$ direction is alleviated, there is a falling off that occurs in the 3–0.3 Hz frequency band. In the 10–0.3 Hz frequency band, the series of sharp falls in the $R_{yx}$ direction are alleviated, but in the 1–0.3 Hz frequency band, the frequency of the $R_{yx}$ direction is obviously dispersed, showing a disjointed trend. Compared with the phase curve of the original data, the frequency of the $P_{yx}$ direction in the 1–0.3 Hz frequency band is still disorderly and discontinuous. 

The red circles are the apparent resistivity-phase curve after being processed by the proposed method. Comparing the original data and StOMP overall processing, the apparent resistivity-phase curve becomes smooth and continuous as a whole because the proposed method retains the low-frequency slow-change component without interference while removing noise. 

In Figure 5, the black diamonds are the apparent resistivity-phase curve of the original data. In the 40–0.3 Hz frequency band, the apparent resistivity curve in the $R_{xy}$ direction asymptotically rises to nearly 45°. In the 2–0.3 Hz frequency band, the apparent resistivity curve in the $R_{yx}$ direction is disorderly and shows a downward trend. In the $P_{yx}$ direction, the phase curve below 10 Hz shows that the phase of some frequency is close to ±180° and the data at these frequencies is completely distorted.

The blue triangles are the apparent resistivity-phase curve after being processed by the StOMP algorithm. In the 40–0.3 Hz frequency band, compared with the original data, it improved to a certain degree in the $R_{xy}$ and $R_{yx}$ directions, and the apparent resistivity value is relatively stable. However, in the 3–0.3 Hz frequency band, the falling off of the curve in the $R_{xy}$ and $R_{yx}$ direction is obvious, and the scattered points in the phase of the $P_{yx}$ direction increase.

The red circles are the apparent resistivity-phase curve after being processed by the proposed method. In comparison to the original data and StOMP overall processing, the apparent resistivity curve of the proposed method is smooth and continuous, and has the least number of scatter points. 

### 4.3. Polarization Direction Analysis

**Figure 6:**
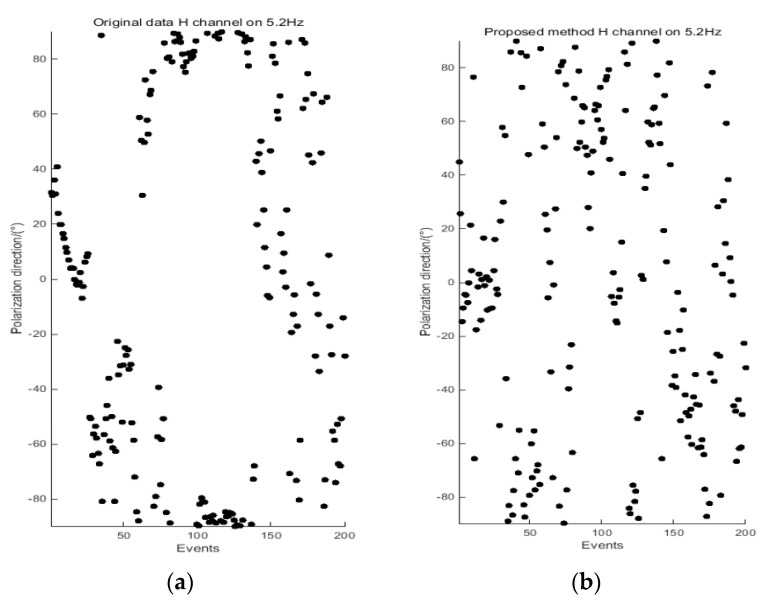
Comparison of the polarization direction results at 5.2 Hz for site 2535BOAC, derived from the original data (**a**) and data processed by the proposed method (**b**).

**Figure 7:**
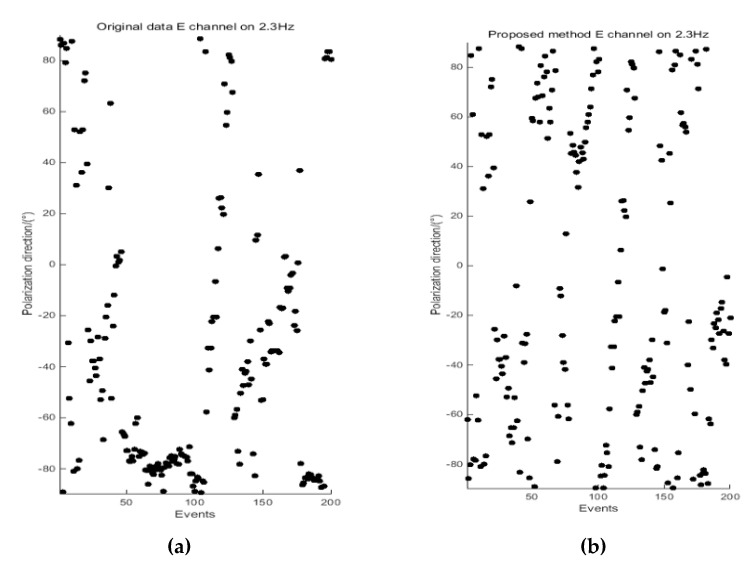
Comparison of the polarization direction results at 2.3 Hz for site 2535BOAF, derived from the original data (**a**) and data processed by the proposed method (**b**).

The polarization direction [52] of the electromagnetic field is one of the important indicators used to evaluate the degree of interference. To further verify the effectiveness of the proposed method, the polarization direction of the electromagnetic field is calculated and analyzed. Figure 6 and Figure 7 present a comparison of the polarization direction results at 5.2 Hz for site 2535BOAC, and at 2.3 Hz for site 2535BOAF, respectively.

According to the definition of the polarization direction of the electromagnetic field, the polarization direction is random when there is no strong interference. Otherwise, the polarization direction will be relatively concentrated and regular. As shown in Figure 6, the polarization direction of the H channel (magnetic field) is concentrated between 70°and 90°, and in Figure 7, the polarization direction of the E channel (electric field) is concentrated between −60°and −90°. The reason is that different types of strong electromagnetic interference affect the original data. By analyzing the polarization directions of the data processed by the proposed method, the polarization directions of the electric and magnetic fields are slowly scattered to different directions, which is close to the random characteristics of the natural electromagnetic field. Combined with the apparent resistivity-phase of these two measured sites at 5.2 Hz and 2.3 Hz in Figure 4 and Figure 5, we can see that the data quality is greatly improved, preserving more of the useful and reliable underground geoelectric information.

## 5. Discussion

With the development of society and economy, the demand for energy is increasing. To meet this demand, we must extend our research efforts to deep underground, using existing technology to analyze collected data to find more energy and resources. However, various electromagnetic interferences have also increased, especially in the eastern part of China, where a dense population, many high-voltage electric wires, communication towers, and highways, etc. severely restrict the reliability of magnetotelluric data. To obtain more useful and reliable magnetotelluric data, we urgently need to find a more effective treatment. Most existing methods include the overall processing of magnetotelluric data, and they lack signal-noise identification. Most importantly, the interference of many measured sites is not particularly dense. Thus, the overall processing of the magnetotelluric data is not necessary to avoid the loss of useful information. By observing the time domain waveform of the measured magnetotelluric data, this can finally be processed in a targeted manner, which preserves more low-frequency slow-change information for better analysis of the magnetotelluric data. 

ApEn and MSE analyses can quantitatively describe the randomness or irregularity of the magnetotelluric signal and both have good noise robustness. The experimental verification showed that the ApEn combined with MSE, used to describe magnetotelluric signals, has obvious advantages. Figure 1 demonstrates the above points. Two kinds of entropy and k-means clustering were introduced here to avoid the over-processing of the signal caused by the overall processing and to thereby avoid losing the important information needed. Figure 2 and Figure 3 illustrate the above. To perform effective signal-noise separation on magnetotelluric data, we introduced the StOMP algorithm, and Figure 2, Figure 3, Figure 4 and Figure 5 illustrate the effectiveness of the de-noising. The information of the low-frequency electromagnetic field can truly reflect the underground distribution information. In addition, we only processed data that were identified as containing strong interference. The effectiveness of the method is directly reflected in the effectiveness of the low-frequency data processing and the similarity to the characteristics of the natural magnetotelluric signals. Figure 4, Figure 5, Figure 6 and Figure 7 illustrate the problem mentioned above.

The application of the proposed method is based on artificial segmentation of the magnetotelluric data without adaptive segmentation based on the length of the interfered signal, so it may cause over-processing of small amounts of data. The StOMP algorithm is required to set the threshold to update the selected set of atoms for each iteration. However, it is especially difficult to select this parameter in actual operation. Therefore, in future research, intelligent algorithms, such as particle swarm optimizations, artificial fish swarm algorithms, and ant colony algorithms, will be introduced to solve the adaptive segmentation of magnetotelluric data and the threshold selection in the StOMP algorithm.

## 6. Conclusions

Natural magnetotelluric signals are extremely weak and irregular relative to a wide variety of strong interferences. How to identify signal and noise, and extract useful magnetotelluric signals from strong interference has become an inevitable problem in the field of magnetotelluric sounding. When faced with massive amounts of measured magnetotelluric data, the distinction of different signals by ApEn and MSE is still very obvious. Two types of entropy were input to k-means clustering for signal-noise identification of the magnetotelluric signal, and were used to accurately identify strong interference and useful signals. Signal-noise separation with the StOMP algorithm was possible only for data segments identified as containing strong interference. Therefore, the proposed method effectively ensured identification accuracy and improved de-noising performance. Experiments showed that the apparent resistivity-phase curve is more continuous and smooth after being processed by the proposed method, which better preserves the low-frequency slow-change information of the magnetotelluric signal and greatly improves the data quality. The magnetotelluric data processed by the proposed method will provide important geoelectric information for subsequent geological inversion interpretation.

## Figures and Tables

**Table 1:** De-noising performance.

Type of Interference	NCC	SNR
Square wave interference	0.9775	13.5165
Triangular wave interference	0.9610	11.5246
Pulse interference	0.9852	15.3308
